# Pulmonary Tuberculosis and Associated Factors Among Diabetic Patients Attending Hawassa Adare Hospital, Southern Ethiopia

**DOI:** 10.2174/1874285801812010333

**Published:** 2018-10-18

**Authors:** Ademe Abera, Gemechu Ameya

**Affiliations:** 1 Hawassa Comprehensive Specialized Hospital, College of Medicine and Health Sciences, Hawassa University, Hawassa, Ethiopia; 2 Department of Medical Laboratory Science, College of Medicine and Health Sciences, Arba Minch University, Arba Minch, Ethiopia

**Keywords:** Associated factors, Diabetic mellitus, Pulmonary tuberculosis, Ethiopia, HIV seronegative

## Abstract

**Background:**

Developing countries have a high burden of Tuberculosis (TB); although it is considered as a disease of the past in most developed countries. The end TB strategy was predicted to stabilize or drop the incidence of TB. However, the rising of the prevalence of immune-related diseases like Diabetes Mellitus (DM) are challenging the TB control strategy in high TB burden region. The objective of this study was to determine the prevalence and associated factors of pulmonary tuberculosis in DM patients attending Adare Hospital, south Ethiopia.

**Methods:**

A cross-sectional study was carried out on 207 randomly selected diabetic patients at the Adare hospital. A structured pre-tested questionnaire was used during the data collection from participants. Sputum concentration technique followed by Ziehl-Neelsen staining method was used to examine pulmonary tuberculosis. Logistic regression analysis was used to assess the association between various variables and pulmonary tuberculosis. Odds ratios and 95% CI were computed to determine the strength and presence of the association.

**Results:**

The prevalence of pulmonary tuberculosis among diabetics was 5.3% [95% CI: (2.2, 8.4)]. Diabetic patients who were underweight [AOR = 9.94, 95% CI: (1.51-80.89)], had more than 10 years duration with DM [AOR = 7.03 95% CI: (1.357, 73.6)], Alcohol drinking habit [AOR = 12.49, 95% CI: (3.28, 77.94)], and history of contact with TB [AOR = 5.35, 95% CI: (1.1-39.12)] were the factors positively associated with pulmonary TB infection while being HIV seronegative had a negative association with pulmonary TB infection [AOR =0.074, 95% CI: (0.001-0.29)].

**Conclusion:**

High proportion of pulmonary TB was observed in diabetic patients as compared to the national estimated prevalence of TB in the total population. Duration of patient with DM, being underweight, alcohol consumption habit, and contact history with TB were positively associated with pulmonary TB infection while being HIV seronegative had a negative association with the infection in diabetic patients.

## 1 BACKGROUND

Tuberculosis is the major public health problem. Different efforts are undertaken to control TB infection, but it is still the major cause of mortality and morbidity in developing courties like Ethiopia. In 2014 global report, there were 9.6 million people who developed active tuberculosis and 1.5 million death cases were reported. Ethiopia which was one of the high burden TB countries met the 2015 WHO target for incidence, prevalence and mortality, even though Africa has still the high burden of TB per total population. According to the World Health Organization (WHO) 2015 report, Africa was the most affected continent by TB which accounts 281 per 100, 000 people while global average TB case report was 133 per 100, 000 people. Worldwide, about 20% of previously treated TB cases and 3.3% of new cases have multi-drug resistance TB according to the WHO report [1].

Only ten percent of *Mycobacterium tuberculosis*-infected people will develop active pulmonary disease in their lifetime. Diabetics and people suffering from other chronic diseases have a much higher incidence of disease than the general population [2]. Diabetes Mellitus is a chronic metabolic disease which occurs when the human body does not respond to the insulin that is produced insulin or when the body is not able to produce enough insulin. Worldwide, people suffering from diabetes are estimated to be 422 million with the prevalence of 8.5% among over 18 years of age according to 2014 WHO report. Its prevalence has been rising more rapidly in developing countries [3]. There were over 1.33 million cases of diabetesc cases in Ethiopia according to the 2015 international diabetic federation report [4].

The association between Diabetes Mellitus (DM) and tuberculosis has been documented long time ago. Their association can be the next challenge for global tuberculosis control. Enhanced understanding of the bidirectional relationship of diabetics and tuberculosis is necessary for proper planning and collaboration to reduce the dual burden of the diseases. Developing countries are still combating with tuberculosis and DM [5]. The incidence rate of TB is very high in sub-Saharan Africa in which Ethiopia islocated . According to 2015 WHO global TB report, prevalence and incidence of TB cases were estimated to be 200 and 207 per 100 000 people, respectively [1].

Diabetes mellitus impairs immune function that specifically affects cell mediated immunity, has been associated with low levels of polymorphoneutrophils and reduce the cytokine level of T-helper1 response to TB. In diabetes, macrophage function is inhibited, phagocytic and chemotactic abilities are impaired despite the immune responses being important for tuberculosis inhibition. Therefore, compromised immunity due to diabetes mellitus would conceivably gives an opportunity for TB development [6]. In the coming decades, the end TB strategy predicted to stabilize drop incidence of TB in most parts of the world. Therefore, the rising burden of diabetes has a relative contribution to the high prevalence of TB in developing countries [7-9].

Diabetes Mellitus increase the risk .developing latent and active tuberculosis infection, and it complicates the treatment of active TB [8]. It is difficult to treat TB infection in the presence of poor glucose control. On the other hand, delayed sputum culture conversion affects the diagnosis, it also increases the case fatality rate of the patient on treatment and relapse rate after the successful completion of treatment. On the other hand, a study showed that TB treatment reduced the efficiency and concentration of diabetes medications, which results in complicatedness in disease control [8, 10, 11]. In Ethiopia now a day, most health centers provide Acid-Fast Bacilli (AFB) stain of sputum smear for pulmonary TB diagnosis, whilst culture is carried out in limited laboratories [12].

In a previous study, prevalence and factor associated with TB infection among DM patients have been documented [13-16]. However, little is known about the epidemiology of pulmonary TB among diabetic people in Ethiopia. There is no study conducted on pulmonary TB among diabetic patients in southern Ethiopia where this study was conducted. Therefore, the aim of this study is to determine the magnitude and associated factors of pulmonary tuberculosis in diabetic patients attending Adare Hospital, southern Ethiopia.

## 2 METHODS

### 2.1 Study Settings and Design

A cross-sectional study was conducted to assess the prevalence of pulmonary tuberculosis and associated factors in diabetic patients attending Hawassa Adare hospital, southern Ethiopia. Hawassa is located 275 km to the south of Addis Ababa. It is the capital city of the Southern Nations, Nationalities, and Peoples Regional state. According to the 2007 census report, the region has a total population of 15,321,000 [17]. The hospital is giving clinical service for more than half a million people in its districts. There were 800 diabetic patients who monitor their disease status in the hospital. Diabetic patients with clinical indications of TB are examined and those found infected are also managed in the TB and leprosy clinic in the hospital. The study period was from March to May 2015.

### 2.2 Study Population

Diabetic patients who were on follow-up in Adare hospital and who had the registration were included in the study. Then people with full information and on follow-up for DM who were above 15 years old were included. However, those who attained diabetic clinic for less than a month, critically ill patient and kyphoscoliosis (difficult to measure height) patient were excluded from the study.

### 2.3 Sample Size and Sampling Procedures

The sample size was calculated using single population proportion formula: n = (Zα/_2_)^2^*p(1-p) /w^2^, by considering the following assumptions: the proportion (p = 0.062) was taken from the previous study [18-20] of prevalence of TB among diabetic patients in the previous study which was 6.2%, 3% marginal error and 10% non-response rate was taken. Therefore, the total number of the sample size was 207.

Systematic random sampling technique was employed to sample the patients using unique diabetic follow-up number. The subject participated in the study was selected by K^th^ number of patients. There were about 800 diabetic patients attending the hospital. Thus: K= 800/207 ≈ 4. Therefore, the study participant was selected in every 4 patients according to the diabetic’s unique numbers of their registration book.

### 2.4 Data Collection Tools and Procedures

Data collection tool was developed after reviewing the literature and all the potential variables related to the objective of the study were included [5, 6, 8, 13-16, 21-32]. The data collection tool was tested on 5% of similar study participants before being used in another health facility which had a diabetic clinic to modify the instrument. The questionnaire was designed to obtain information related to the socio-demographic characteristics of study participants, behavioral, nutritional, and medical characteristic. Nutritional status of patients on diabetic follow up was assessed by measuring weight and height of the participants.

### 2.5 Specimen Collection and Laboratory Investigations

Diagnosis of pulmonary tuberculosis among diabetic patients was done according to the national TB diagnosis guideline [19]. All participants provided three consecutive sputum samples (spot-morning-spot) using dry, leak-proof and clean container. To enhance the detection rate of AFB, bleach concentration technique was used according to the procedure explained in part two of Cheesbrough M [20]. Then the samples were stained using the Ziehl-Neelsen staining method for the diagnosis of AFB. TB positive sample was reported as positive if at least two AFB smear results were positive or one smear was positive with x-ray finding [19].

### 2.6 Quality Control

Internal quality control was done according to AFB smear microscopy manual [19]. Staining reagents were tested by known negative and positive control slides. All slides were read by experienced medical laboratory technologist, and the entire positive and 10% of AFB negative slides were blindly re-checked by other qualified professionals.

### 2.7 Ethical Approval and Consent to Participate

Institutional ethical clearance was taken from research and ethics review board of Hawassa University. An official letter to obtain permission from the medical director of Hawassa Adare hospital was also written from the University before the data collection. The written consent and information about the purpose and importance of the study were given to each participant before the data collection. Privacy of the participants was secured at every point of the study. Patient participated in the research was on voluntary bases and those who were requested to quit their participation at any stage of the study were clued-up to do so without any limit.

### 2.8 Data Processing and Analysis

The gathered data were entered to Epi-Info version 3.5.1 and transferred to SPSS version 20.0 for additional analysis. Logistic regression analysis was used to assess the association between various variables and pulmonary tuberculosis. Odds ratios and 95% CI were computed to determine the strength and presence of the association.

## 3 RESULTS

### 3.1 Socio-Demographic Characteristics of the Participants

In the study, 207 diabetic patients participated with zero non-response rate. More than half of the participants were females 107 (51.7%). The age of participants ranged from 17 to 95 years and the mean age was 48.7 years with ±11.5 standard deviation. The majority of participants were married 168 (81.2%) and urban dwellers 180 (87.0%). Regarding the educational status of participants, 66 (31.9%) of them were illiterate and 49 (23.7%) of them were educated up to the level of college and above. Nearly half of the participants 93 (44.6%) were Orthodox religion followers followed by protestant religion which accounts about 34% of participants. Regarding the occupational status of the participant, 96 (46.4%) of the participants were employed (government or private) in contrast, 87 (42%) of them were unemployed. Monthly income of participants was also assessed and about 42% of the participants got less than 500 Ethiopian Birr per months and only about 10% of participants got more than 2000 Birr per month. More than two third of the study participants had 5 to 10 family member (Table **1**).

### 3.2 Medical Characteristic of DM Patients

Of 207 participants, 171 (82.61%) of them were with type two diabetes mellitus while the rest were type one. All patients had regular follow-up either treated by pills or insulin treatments. More than half 111 (53.6%) of the participants were taking pills while The others were taking insulin as treatment. Duration of participants with DM after diagnosis ranged from 1 year to 27 years and the mean duration was 7.15 with 5 years standard deviation. Furthermore, the majority of the participants 84 (40.6%) were with DM for less than five years after diagnosis while about 25% of them were living with the disease for more than 10 years. Of participants, 10 (4.8%) of them were HIV positive. Regarding the body mass index of the participants, majority of 130 (62.8%) were over-weight while only 17 participants were under-weight.

### 3.3 Smear Positive Pulmonary TB in Diabetic Patients

**Fig. (1):**
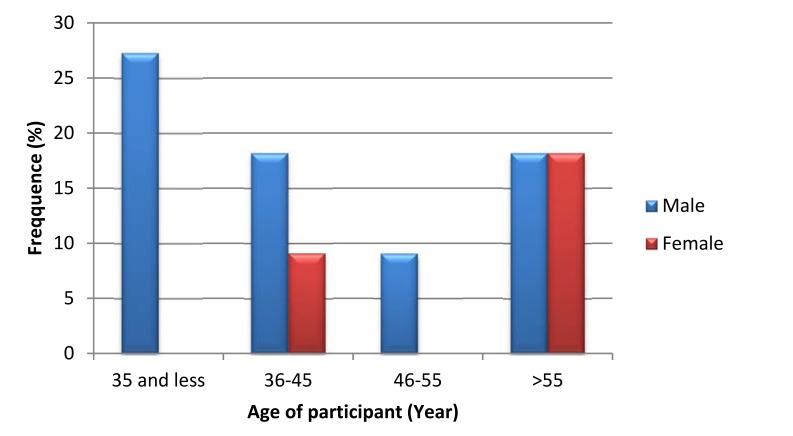
Smear positive pulmonary tuberculosis by age and sex among diabetic patient in Hawassa Adare Hospital, Southern Ethiopia.

Out of 207 TB screened diabetic patients, 11 (5.3%) [95% CI: (2.2-8.4)] of them were smear-positive pulmonary TB by sputum concentration acid-fast stained direct microscopy technique. Of smear-positive patients, majority of them (72.7%) were male patients and above fifty-five years old. Similarly, majority of them were married participants. A relatively highest proportion of smear-positive pulmonary tuberculosis was observed in age groups less than 35 years which accounts for about 15.78% (3/19) of the age group followed by age group older than 55 years with 6.4% (4/62) (Fig. **1**). Proportion of smear positive pulmonary TB was almost similar in all educational statuses of participants but slightly high proportion was observed in participants who educated up to college and above. High proportion of pulmonary tuberculosis was exhibited in a participant who had more than ten family members per household than less family member. Proportion of tuberculosis was also determined in terms of income of study participants and high proportion was observed in patients with less than one thousand Ethiopian Birr per month. Relatively high proportion of smear positive pulmonary tuberculosis was detected among students followed by unemployed participants.

### 3.4 Factor Associated with Smear-Positive Pulmonary Tuberculosis in Diabetic Patients

In the bivariate analysis of logistic regression, statically significant association was observed between outcome variable and religion, duration of DM with patient, Body Mass Index (BMI), alcohol consumption habit, smoking habit, type of treatment for DM, contact history with TB, and HIV status of participants. In multivariable analysis, all socio-demographic characteristics of participants, smoking habit, and type of treatment for DM were insignificant. But, the duration of DM with patient, BMI, alcohol consumption habit, contact history with TB, and HIV status of participants were significantly associated with pulmonary tuberculosis among diabetic patients.

Diabetic patients who were underweight [AOR = 9.94, 95% CI: (1.51-80.89)] were about ten times more likely infected with pulmonary tuberculosis as compared to those who were overweight. Those patients living with DM for more than 10 years after DM diagnosed [AOR = 7.03 95% CI: (1.357, 73.6)] were seven times more likely to have pulmonary tuberculosis as compared to those living with DM for less than five years. Alcohol consumption habit of diabetic patients revealed the odds of infection with pulmonary tuberculosis by about twelve times higher among alcohol drinkers than those who were non-drinkers [AOR = 12.49, 95% CI: (3.28, 77.94)]. Diabetic patients who had contact history with tuberculosis [AOR = 7.35, 95% CI: (1.1-39.12)] had about five times higher odds of infection with tuberculosis as compared to those patients without TB contact history. Being not infected with HIV among diabetic patients minimize the chance of being infected with pulmonary tuberculosis by about 93% [AOR =0.074, 95% CI: (0.001-0.29)] (Table **2**).

## 4 DISCUSSION

The overall prevalence of pulmonary TB in diabetics was shown to be 5.3% in our study which was higher than the estimated prevalence of TB in total population [1]. This finding is in line with the studies conducted in Tanzania (5.4%) [22] and India (6%) [23]. Moreover, our finding is also comparable to the study carried out in Dessie, northeast part of Ethiopia (6.2%) [18] and a study conducted in Addis Ababa (5.8%) [24]. However, it is lower than the findings from Pakistan (14%) [25] and South Africa (10.6%) [13]. In contrast, our finding is higher than the finding of a study conducted in India (2.6%) [26]. The observed difference in the pulmonary tuberculosis prevalence among diabetic patients in Pakistan might be due to the difference in the severity of diabetic patient which were admitted in the study while in South Africa, they also use the radiographic diagnostic method. Moreover, the difference with the low prevalence of tuberculosis in Indian diabetic patients might be due to the difference in socio-demographic characteristic of the population as well as due to the difference in detection ability of the diagnostic methods.

Patients living with DM are at high risk of developing active tuberculosis which may impair both innate and adaptive immune responses to *Mycobacterium tuberculosis*. The relationship between neutrophils and autoimmune diabetes has also been documented. The number of macrophages is low in diabetes as compared non-diabetes and macrophages are important for the activation of the cellular immune response [6]. The increased risk of TB associated with diabetes may be also attributed to the chronic nature of the illness and failure to control glucose that may enhance the risk of TB [5, 10, 21].

In the current study, duration of patient with DM was associated with the occurrence of pulmonary tuberculosis infection in diabetic patients. Those diabetic patients live with DM for more than ten years after they had been diagnosed for DM had seven times high odds of infection with TB than those patient living with DM for less than five years [AOR = 7.03 95% CI: (1.357, 73.6)]. This finding is similar to a study conducted in Dessie, Ethiopia [AOR: 8.89; 95% CI: (1.88-58.12)] [18] and a study conducted in Pakistan [14]. This may be due to the complication of DM when in long duration. The immunity of the patient is also more compromised ina patient living with DM for a long time as a result of microvascular disease and macrovascular disease as well as treatment tolerance of the disease.

The other finding of the current study revealed that those DM patients who were underweight (BMI <18.5Kg/m^2^) had about ten times high odds infection with DM as compared to those with body weight gains [9.94, 95% CI: (1.51, 80.89)]. Similar finding was obtained in a study conducted in Chiayi, Taiwan [OR = 6.635, 95% CI: (2.096-21.007)] [15], a study conducted in India [AOR = 2.03, 95% CI: (1.32–3.12)] [16] and in Tanzania [OR = 2.08 955 CI: (1.06– 4.06)] [27]. BMI indicates malnutrition which is a factor for a number of infectious diseases. Underweight individuals have a weak immunity system which expose them to infection.

In the present study, there was a statically significant relation between alcohol consumption habit and pulmonary tuberculosis in diabetics. A Study conducted in Taiwan also showed that consumption was an associated factor for tuberculosis infection in male diabetics [OR = 2.410, 95% C: (1.425-4.074)] [15]. The other study conducted in India also showed an association between alcohol consumption and TB infection [28]. A systematic review also indicated the association between alcohol addiction and tuberculosis [29]. A number of reasons have been documented concerning their association. One of these reasons is the toxic effects of alcohol on the immune system making the host more susceptible to TB infection. In animal studies, the chronic and acute alcohol consumption directly impaired cell-mediated immunity and macrophage functions [29].

Contact history of diabetic patients with TB had a significant association with pulmonary tuberculosis; those diabetic patients who responded to their contact with TB infected person had about five times high odds of infection with TB [AOR = 5.35, CI: (1.1, 39.12)]. Similar finding was observed in a study conducted in Dessie, Ethiopia [(AOR = 9.4; 95% CI: (1.822-48.50)] [18]. Systematic review also showed that contacts with tuberculosis patients are a high-risk group for developing TB [30]. Even though some studies showed the prevalence of latent TB infection and TB among contacts is significantly less in developed countries, diabetic patient’s contact with TB has a significant association in our study. This might be due to the impaired immune system in diabetic patients that makes them susceptible to various infections [30]. Being HIV positive was also associated with pulmonary TB infection in diabetics in this study. Similar finding was observed in a study conducted in rural south Indian [31] and in Ethiopia [32]. Another study conducted in India also showed a strong association among TB, diabetics and HIV [33].

Social desirability bias and recall bias are the potential limitations of this study. The lack of sufficient study in Ethiopian context made the comparison of the results difficult. Even though direct microscopic examination is not a gold standard method, it is highly utilized in the diagnosis of pulmonary tuberculosis in developing countries. It is a less sensitive method and it may underestimate the pulmonary TB prevalence unless it is supported by sputum concentration method. In our study to modify its sensitivity, we conducted the direct AFB stain with Sputum concentration technique.

## CONCLUSION

Prevalence of pulmonary tuberculosis in the diabetic patient was high as compared to the national estimated prevalence of TB in the total population. Duration of patients with DM after they have been diagnosed, being underweight, alcohol consumption habit, and contact history with tuberculosis were positively associated with pulmonary TB infection while being HIV seronegative, they had been negatively associated with pulmonary tuberculosis infection in diabetic patients. By considering these findings it is recommended that TB control programs, policy makers and stakeholders should increase their efforts.

## Figures and Tables

**Table 1:** Socio- demographic characteristics of diabetic patient in Hawassa Adare Hospital, Southern Ethiopia, from March to May 2015 (n=207).

Variable	Category	No. (%)
Sex	Male	100 (48.3)
	Female	107 (51.7)
Age in years [Mean: 48.7 years]	< 35	18 (8.7)
	36-45	74 (35.7)
	46-55	53 (25.6)
	>55	62 (30.0)
Marital status	Single	29 (14.0)
	Married	168 (81.2)
	Widowed	10 (4.8)
Religion	Catholic	8 (3.8)
	Muslim	35 (16.9)
	Protestant	71 (34.3)
	Orthodox	93 (44.9)
Educational status	Not able to read	66 (31.9)
	Primary school	45 (21.7)
	Secondary school	47 (22.7)
	College and above	49 (23.7)
No. of family	<5	58 (28.0)
	5-10	142 (68.6)
	>10	7 (3.4%)
Resident	Urban	180 (87.0)
	Rural	27 (13.0)
Monthly income [Ethio. Birr]	<500	87 (42.0)
	501-1000	70 (33.8)
	1001-2000	28 (13.5)
	>2000	22 (10.6)
Occupational status	Employee	96 (46.4)
	Student	4 (1.9)
	Merchant	7 (3.4)
	Farmer	13 (6.3)
	Unemployed	87 (42.0)

**Table 2:** Factor associated with pulmonary tuberculosis among diabetic patient in Hawassa Adare Hospital, Southern Ethiopia, 2015 (n=207).

**Variables**	**TB status**		**Odds ratio (95% CI)**		**P-Value**
	**Smear Positive**	**Smear Negative**	**Crude**	**Adjusted**	
**Duration with DM (In year)**					
**<5**	2	82	Ref.	Ref.	
**6-10**	3	67	1.836 (0.29, 11.31)	11.62 (0.89, 51.63)	0.061
**>10**	6	47	5.23 (1.02, 26.98)	7.03 (1.357, 73.6)	0.028
**Body mass index (Kg/m^2^)**					
**<18**	3	14	6.75 (1.37, 33.29)	9.94 (1.51, 80.89)	0.024
**18-24.5**	4	56	2.25(0.54, 9.32)	7.09 (0.54, 53.16)	0.136
**>24.5**	4	126	Ref.	Ref.	
**Alcohol consumption habit**					
**Yes**	8	23	10.58 (2.96, 41.05)	12.49 (3.28, 77.94)	0.002
**No**	3	173	Ref.	Ref.	
Smoking habit*					
**Yes**	7	11	29.47(7.47, 115.91)	4.81 (0.29, 59.04)	
**No**	4	185	Ref.	Ref.	
Type of treatment*					
**Pills**	3	108	0.31 (0.079, 1.19)	0.29 (0.039, 2.23)	
**Insulin**	8	88	Ref.	Ref.	
**Contact history with TB**					
**Yes**	7	19	7.303 (3.37, 40.81)	5.35(1.1, 39.12)	0.04
**No**	4	177	Ref.	Ref.	
**HIV status**					
**Negative**	7	190	0.055 (0.013, 0.241)	0.074 (0.001, 0.29)	0.006
**Positive**	4	6	Ref.	Ref.	

*=Not significant in backward stepwise logistic regression; Ref.: Reference

## LIST OF ABBREVIATIONS

AFB: = Acid Fast Bacilli
AOR: = Adjusted Odd Ratio
BMI: = Body Mass Index
CI: = Confidence Interval
DM: = Diabetic Mellitus
HIV: = Human Immunodeficiency Virus
SPPTB: = Smear Positive Pulmonary Tuberculosis
TB: = Tuberculosis
WHO: = World Health Organization
